# Effectiveness of a spray containing 1% malic acid in patients with xerostomia induced by graft-versus-host disease

**DOI:** 10.4317/medoral.22699

**Published:** 2019-03

**Authors:** Elena Bardellini, Francesca Amadori, Giulio Conti, Federica Veneri, Alessandra Majorana

**Affiliations:** 1 AP, DDS, MS. Department of Pediatric Dentistry, Dental Clinic, University of Brescia Brescia, Italy; 2 DDS PhD. Department of Pediatric Dentistry, Dental Clinic, University of Brescia Brescia, Italy; 3 DDS, MS, PhD University of Milan, Milan, Italy; 4 DDS. Department of Pediatric Dentistry, Dental Clinic, University of Brescia Brescia, Italy; 5 FP, DDS, MS. Department of Pediatric Dentistry, Dental Clinic, University of Brescia Brescia, Italy

## Abstract

**Background:**

To evaluate the clinical effectiveness of a topical sialogogue spray (malic acid, 1%) in the treatment of xerostomia in patients with chronic Graft versus Host Disease (cGVHD).

**Material and Methods:**

This study was designed as a randomized double-blind clinical study. Twenty-eight patients with cGVHD suffering from xerostomia were divided into 2 groups: the first group (14 patients) received a topical sialagogue spray containing malic acid 1% (SalivAktive®) whereas the second group (14 patients) received a placebo. Both groups received treatment for 2 weeks. Dry Mouth Questionnaire (DMQ) scores and unstimulated salivary flows rate were collected before and after treatment.

**Results:**

DMQ scores increased significantly from 1.3 ± 0.4 to 3.5 ± 0.4 points (*p* <0.05) after two weeks of treatment with malic acid, whereas in the control group DMQ scores increased from 1.2 ± 0.7 points to 1.4 ± 0.6 (*p* >0.05). The unstimulated salivary flow rate in patients treated with malic acid increased significantly from 0.15 ± 0.06 mL/min to 0.24± 0.08 mL/min, while that of the patients treated with placebo went from 0.16 ± 0.07 mL/min to 0.17 ± 0.09 mL/min (*p* >0.05).

**Conclusions:**

Malic acid 1% spray can be considered effective in the treatment of GVHD induced xerostomia.

** Key words:**Xerostomia, malic acid, transplantation.

## Introduction

Graft versus host disease (GVHD) is one of the most frequent and serious complications of hematopoietic stem cell transplantation. After transplantation, the donor’s T lymphocytes may recognize the antigens expressed by the re¬cipient’s cells as foreign, inducing an immune reaction accompanied by intense inflammatory responses. The acute presentation of GVHD typically affects the skin, the gastrointestinal tract, and the liver. The chronic form is characterized by the involvement of several organs, including the oral cavity, which could represent the primary site of chronic GVHD (cGVHD) (1-3).

The prevalence of oral cGVHD ranges between 45-83%; moreover, the oral cavity could be the only affected body region (4-5). The specific or distinctive clinical oral features comprise oral lichenoid lesions, hyperkeratotic plaques, mucosal atrophy, erythema, ulcers, pseudomembranes, limited oral aperture secondary to sclerosis and, last but not least, xerostomia.

Xerostomia can manifest itself with some frequency as a consequence of progressive salivary gland damage. Salivary gland dysfunction in cGVHD patients mimics Sjögren syndrome; it is characterized by histopathological changes, consisting of mononuclear infiltration with periductal infiltration, and/or atrophy of salivary gland lobules and peri-glandular fibrosis (6). Recent studies reported that saliva in patients with cGVHD have significantly higher concentrations of sodium, magnesium, epidermal growth factor, total protein, albumin and immunogoblin G (7). As a result of the histopathological changes and of the altered salivary concentrations, the functional capacities of saliva (i.e. chemical digestion; lubrification and cleaning; pH buffering effect; anti-inflammatory, anti-bacterial and anti-viral activity) are impaired. The repercussions are a disruption of the mucosal integrity, insufficient protection against mechanical and chemical epithelial injuries, and reduced anti-cariogenic activity (7). Furthermore, a dry mouth can compromise swallowing, speaking, and sleeping; this, in turn, is associated to a worsening of a patient’s quality of life referred to the oral cavity and due to a diminished body mass index (8).

Although no standard treatment guidelines are available, many treatment options exist for the management of xerostomia and hypo-salivation, from topical agents to systemic therapies. Intraoral topical agents, being free of side effects, are among the most commonly recommended treatments for the management of xeros¬tomia. The purpose of this study was to evaluate the effectiveness of a topical sialogogue spray containing malic acid 1% (Salivaktive®) in patients with cGVHD experiencing xerostomia.

## Material and Methods

-Study population

Thirty-one consecutive patients were enrolled. Inclusion criteria were transplanted subjects who developed cGVHD and dryness in the mouth. Exclusion criteria were head-neck radiation therapy, Sjogren’s syndrome and/or related autoimmune diseases (rheumatoid arthritis, polyarthritis nodosa, systemic sclerosis or lupus erythematosus), diabetes, current xerostomic medication (anti-hypertensives, anti-depressants, anxiolytics, anti-psychotics, anti-cholinergics, adrenergic blockers, anti-asthmatics), smoking and chronic alcohol abuse.

-Study design

This study was designed as a randomized controlled clinical study. Sample size was based on the standard deviation of the main variable: Dry Mouth Questionnaire (DMQ). A pilot sample of 10 patients was tested to determine the minimum sample size necessary to reliably confirm the hypothesis that a topical 1% malic acid sialogogue spray is effective in treating xerostomia induced by cGVHD over a 2 weeks period.

The patients were randomly assigned to two groups. Randomization was performed using an automatically generated list in a 1:1 block size for two patients. The patients included received a number and were randomly assigned to one of the two sprays by a member of the team who performed a permuted-block randomization of the participants. Patients codes where inserted into closed envelopes.

The patients were randomized by a computer code into two groups: group A, who received a topical sialogogue spray (malic acid, 1%) and group B, who received a placebo (i.e. saline solution).

The two products shared the same packaging. The sprays were applied four times a day. Both patients and researchers were blinded throughout the study.

Dry mouth questionnaire (DMQ)

The DMQ was used to assess the severity of dry mouth before and after treatment with malic acid/placebo. (9-11) Every patient answered the DMQ before receiving a spray (1% malic acid or placebo) and after 2 weeks of use. Increased DMQ scores indicate improvement of dry mouth. The DMQ used a 0-to-4 rating scale where 0= very dry and 4=not dry at all ( Table 1).

**Table 1:**
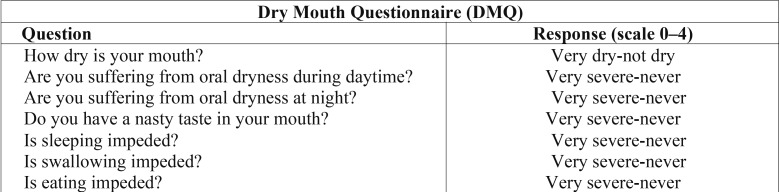
Dry Mouth Questionnaire (DMQ).

-Sialometries

The unstimulated salivary flow rate was obtained by the spit method every 30s for 15 minutes. Saliva was collected in 20-mL plastic containers. Measurements were expressed as millilitre per minute (13).

-Statistical Analysis

The main purpose was to contrast different DMQ scores at T0 and T1 by using the Mann-Whitney U- test. A 5% significance level was used for the analysis. Student’s t-test was used to analyse unstimulated salivary flow rate 

-Ethical Considerations

The research was performed in compliance with the Declaration of Helsinki and Good Clinical Practice. All patients were informed about the research and signed an IRB approved informed consent. Ethical approval for the research was granted by the local Ethic Committee.

## Results

-Characteristics of the patients

**Figure 1:**
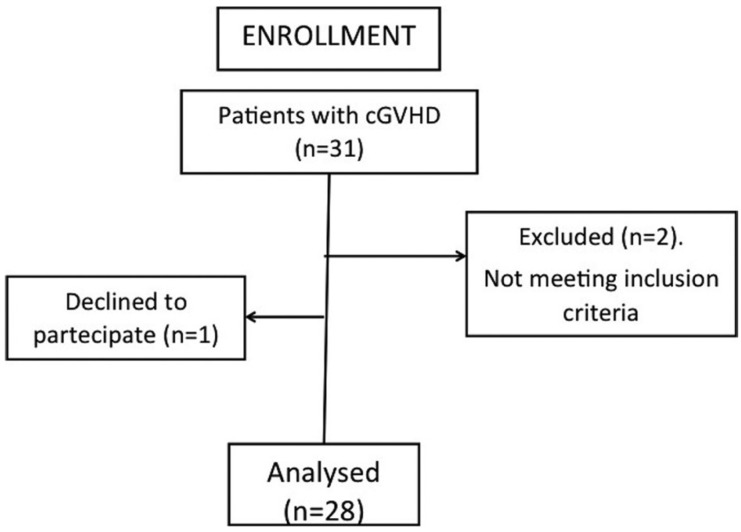
Enrollment flow chart.

Among the 31 patients with cGVHD complaining of xerostomia, 28 met the inclusion criteria (Fig. 1). They were randomly assigned to two groups: group A (n=14, malic acid 1%) and group B (n=14, placebo).  Table 2 shows intervention group versus control group results in relation to age, gender, DMQ scores and unstimulated salivary flow rate.

**Table 2:**
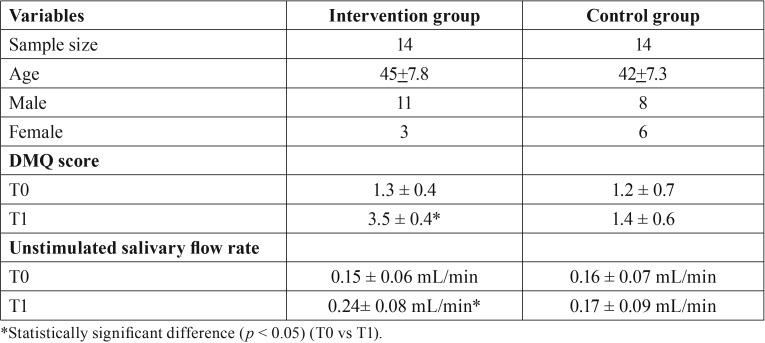
Demographic characteristics of the two groups, DMQ scores and unstimulated salivary flow rate.

There were no statistically significant differences (*p*>0.05) between the demographic characteristics of the two groups; therefore, the two groups were homogeneous.

-DMQ scores

DMQ scores increased significantly from 1.3 ± 0.4 to 3.5 ± 0.4 points (*p*<0.05) after the two weeks treatment with malic acid, whereas in the control group DMQ scores increased from 1.2 ± 0.7 points to 1.4 ± 0.6 (*p*>0.05).

-Unstimulated salivary flow rate

After two weeks of treatment, the unstimulated salivary flow rate increased significantly from 0.15 ± 0.06 mL/min to 0.24± 0.08 mL/min whereas that of the patients treated with placebo went from 0.16 ± 0.07 mL/min to 0.17 ± 0.09 mL/min (*p*>0.05).

## Discussion

Xerostomia, i.e. the subjective sensation of a dry mouth, can be either caused by a hypofunction of the salivary glands (reduced volume of saliva secretion) or by a change in salivary composition. Dry mouth is usually associated with difficulty in swallowing, speaking and numerous other oral health problems. Since 1989 (14), the salivary glands have been recognized as a very sensitive target for cGVHD. Reduction in whole saliva flow rate in GVHD patients has been reported in many studies, as well as various salivary biochemical and immunologic compositional alterations. The mechanism of salivary involvement depends on the lymphocyte infiltration, mostly T cells with a predominance of CD8+ over CD4+, in the glandular parenchyma, especially around the secretor ducts.

Such periductal infiltration associated with a cytokine dysregulation (especially IL-2, IL-6, IFNgamma, TNF alfa) are considered responsible for the salivary parenchymal atrophy and hypofunction (7,15).

This study was carried out on patients with cGVHD complaining of xerostomia. Xerostomia is defined as the subjective complaint of dry mouth. Objective presentation of salivary gland hypofunction does not necessarily reflect a subjective perception of xerostomia and vice versa. Interestingly, patients complaining of xerostomia frequently do not show any objective sign of hypo-salivation and their symptoms may be secondary to qualitative and/or quantitative changes in the composition of saliva. The content and production of saliva by different salivary glands display circadian variations affecting different aspects of symptoms related to salivary dysfunction (16,17).

Xerostomia in patients with objective hypo-salivation is diagnosed when the rate of saliva flow is less than the rate of fluid absorption across the oral mucosa plus the rate of fluid evaporation from the mouth (17,18).

The normal unstimulated salivary flow rate is approximately 0.3–0.4 mL/min (17,19-20).

A diagnosis of hypo-salivation is made when the unstimulated salivary flow rate is around 0.1 mL/min (17,20-21). Our patients had a baseline salivary flow rate ranging from 0.09 to 0.26 mL/min, thus patients with a subjective sensation of xerostomia and those with a hypo-salivation were included.

Although no standard treatment guidelines are available, many treatment options exist for the management of xerostomia in cGVHD. Several treatment strategies have been recommended in the past years and they all aim to reduce patients’ symptoms and/or increase salivary flow. Pilocarpine and cevimeline are two systemic US Food and Drug Administration-approved sialogogues. They are effective, but they have many side effects i.e. excessive sweating, cutaneous vasodilatation, emesis, nausea, diarrhea, persistent hiccup, bronchoconstriction, hypotension, bradycardia, increased urinary frequency, and vision problems (17,22).

Easy remedies are proper hydration, increase in humidity at night-time, avoidance of irritating dentifrices and crunchy/hard foods and use of sugar-free chewing gums/candy.

Currently, intraoral topical agents are among the most commonly recommended treatments for the management of xerostomia. These include saliva stimulants and substitutes (23).

Saliva substitutes aim to increase viscosity and mimic natural saliva without altering the salivary flow. They contain minerals (eg, fluoride, calcium, and phosphate ions), carboxymethylcellulose or hydroxyethylcellulose, flavoring agents, and preservatives (eg, propyl or methyl paraben). Other remedies include mucoadhesive lipid-based bioerodible tablets or mucin sprays, although their efficacy for management of xerostomia remains controversial (17,23).

This study aimed to evaluate the effectiveness of a topical sialogogue spray containing malic acid 1% in patients (Salivaktive®) in patients with cGVHD experiencing xerostomia. The application of topical sialogogues containing acids in the treatment of xerostomia isn’t new. However, continuous application of substances such as citric acid has been related to an increased risk of caries, due to the erosive action of these agents on dentin (24,25).

All these products were dropped because of their demineralising effect on human dentin, effect not only caused by the high doses of acidic products, but also by how the product consumption (chewable products), as some allow for a lengthy contact with the dental surfaces. Products in a spray format allow for a fast and direct contact with the oral mucosa, and this, if combined with a suitable concentration (as the stimulant effect on saliva production is not altered by it), could reduce the demineralising potential of these substances.

Malic acid acts as a sour tasting gustatory stimulus. Its mechanism of action is linked to dissociation of H+ in malic acid in water, which becomes hydronium ions (H3O+); this action generates a stimulation of salivary secretion to dilute the concentration of acids in the oral cavity. Xylitol do not stimulate saliva but it reduces erosion and cariogenic potential. Thus, a spray containing malic acid with xylitol seems to be a safe topical sialogogue (26,27).

DMQ was used as it is an easy and fast method to assess the efficacy of topical sialogogues. In addition to this, its 0-to-4 scale can be easily replaced by a Visual Analogue Scale (VAS) of 10 cm. According to DMQ scores, feelings of oral dryness among intervention group improved significantly in comparison with the control group. The results also showed an increase of the unstimulated salivary flow rate, after the use of 1% malic acid spray for 2 weeks. According to the results of our study, the use of malic acid as a salivary stimulant can be a valid option for the treatment of cGVHD-induced xerostomia.
